# Glucosinolate variability between turnip organs during development

**DOI:** 10.1371/journal.pone.0217862

**Published:** 2019-06-06

**Authors:** Guusje Bonnema, Jun Gu Lee, Wang Shuhang, David Lagarrigue, Johan Bucher, Ron Wehrens, Ric de Vos, Jules Beekwilder

**Affiliations:** 1 Plant Breeding, Wageningen University and Research, Wageningen, The Netherlands; 2 Department of Horticulture, College of Agriculture & Life Sciences, Chonbuk National University, Jeonju, Korea; 3 Wageningen Plant Research, Wageningen, The Netherlands; Chungnam National University, REPUBLIC OF KOREA

## Abstract

Turnip *(Brassica rapa spp*. *rapa*) is an important vegetable species, with a unique physiology. Several plant parts, including both the turnip tubers and leaves, are important for human consumption. During the development of turnip plants, the leaves function as metabolic source tissues, while the tuber first functions as a sink, while later the tuber turns into a source for development of flowers and seeds. In the present study, chemical changes were determined for two genotypes with different genetic background, and included seedling, young leaves, mature leaves, tuber surface, tuber core, stalk, flower and seed tissues, at seven different time points during plant development. As a basis for understanding changes in glucosinolates during plant development, the profile of glucosinolates was analysed using liquid chromatography (LC) coupled to mass spectrometry (MS). This analysis was complemented by a gene expression analysis, focussed on GLS biosynthesis, which could explain part of the observed variation, pointing to important roles of specific gene orthologues for defining the chemical differences. Substantial differences in glucosinolate profiles were observed between above-ground tissues and turnip tuber, reflecting the differences in physiological role. In addition, differences between the two genotypes and between tissues that were harvested early or late during the plant lifecycle. The importance of the observed differences in glucosinolate profile for the ecophysiology of the turnip and for breeding turnips with optimal chemical profiles is discussed.

## Introduction

**Fig 1:**
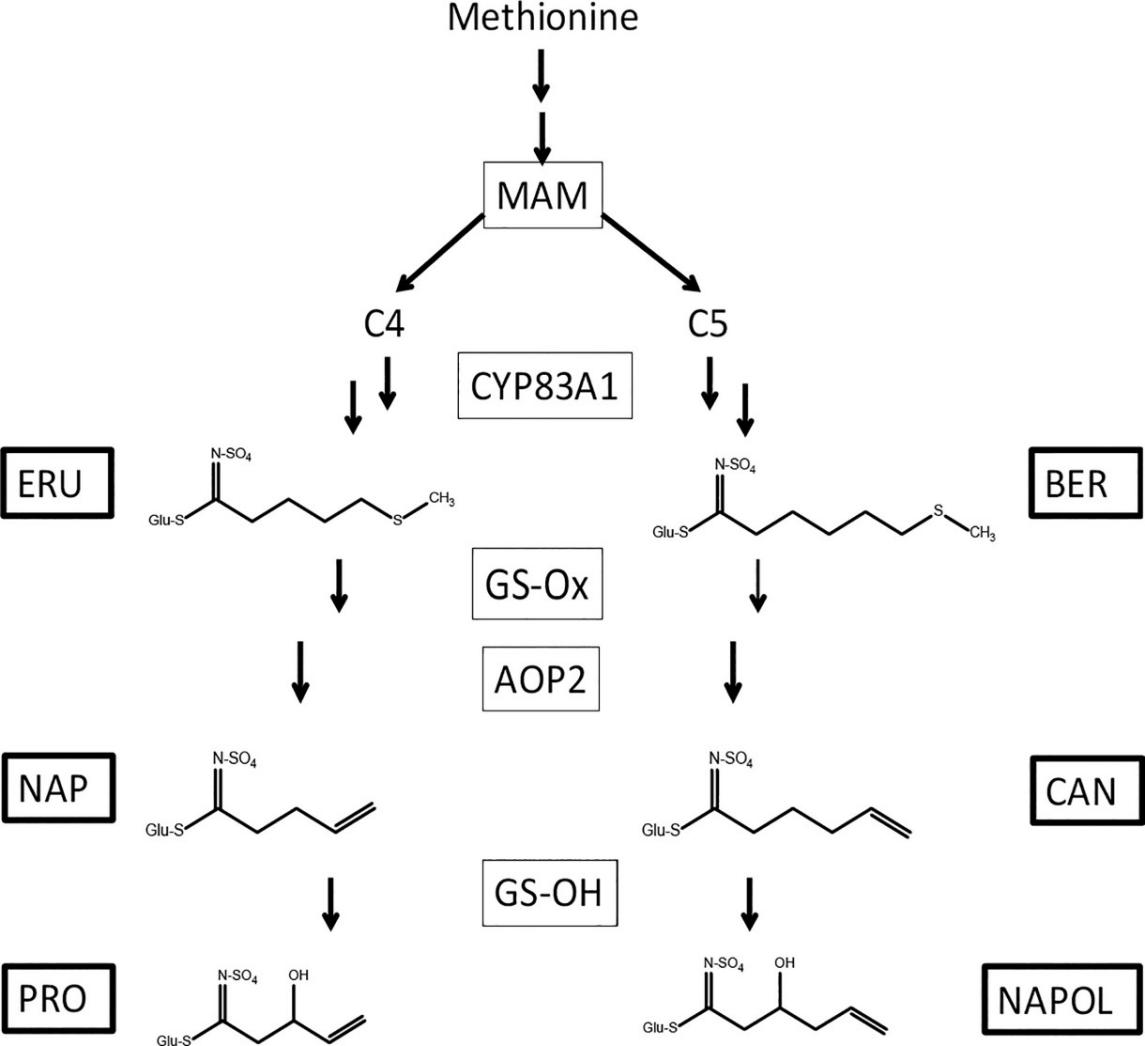
Proposed metabolic pathway from methionine to aliphatic GLS in turnip. GLS compounds are indicated in solid squares and structures of their side-chains are shown; genes are indicated in dashed squares. ERU: glucoerucin (also denoted as 4MTB); BER: glucoberteroin (5MTP); NAP: gluconapin (3-butenyl); CAN: glucobrassicanapin (4-pentenyl); PRO: progoitrin (C42OH); NAPOL: gluconapoleiferin (C52OH).

Turnip *(Brassica rapa* subsp. *rapa*) forms a large and edible tuber, that is composed of both hypocotyl and root tissue [1]. From turnips, both the tubers and green parts are consumed, in particular in temperate regions in Asia, Europe and North America. In addition to its role in human nutrition it is also important as a fodder crop. Turnips are a source of vitamins and nutrients, but also contain significant amounts of glucosinolates (GLS), a group of secondary plant metabolites almost exclusively found in the order Brassicales [2,3,4]. GLS are water-soluble compounds that derive from glucose and amino-acids such as methionine, tryptophan or phenylalanine. The core structure of all GLS consists of thioglucose and sulphate groups, which are conjugated to an amino-acid derived side chain. Side chains can be aliphatic or indolic or aromatic, vary in chain length and can undergo several modifications (Fig 1) [5]. In plants, GLS have a role to protect the plant from insect damage, both in leaves and in underground tissues [6,7,8,9]. In vegetables, GLS provide a variety of tastes like bitterness and pungency. Upon damage to plants, e.g. by chewing, GLS are enzymatically converted into a range of volatile compounds, like nitriles and isothiocyanates (ITCs). In addition to taste formation GLS have been reported to be implicated in both antinutritional and health-promoting effects [10]. Progoitrin, a GLS known from several brassica species, has anti-thyroid activity and promotes goitre disease [11]. On the other hand, a high consumption of Brassica vegetables correlates negatively with the incidence of degenerative diseases in numerous epidemiological studies [11]. Protective effects are often accredited to GLS breakdown products such as ITCs, nitriles and indoles [12,13]. There is increasing evidence that ITCs are involved in cancer prevention and have anti-inflammatory effects (reviewed in [14]).

Previous work has shown that turnip varieties differ widely in their GLS content and composition [4]. Generally GLS composition is measured in either the green parts of turnips [3,15,16], or in the tubers [4,17,18], and these data suggest that the GLS composition of tubers differs strongly from that of leaf tissue. This is likely the result of tissue-specific regulation of GLS biosynthesis, transport and/or storage, which is affected by both the genotype of the plant and the environment [19,20,21,22]. Only recently a single study compared GLS composition in both leaves and tubers of several turnip accessions [23]. In addition, global GLS composition has recently been analyzed in leaves and roots in ecological studies [24].

Breeding towards new turnip varieties with specific GLS composition may be in the interest of consumers in view of their contribution to the nutritional quality of this vegetable. For such breeding activities, it is important to identify genetic loci controlling GLS biosynthesis and storage. In *Arabidopsis thaliana*, discovery of such loci has been strongly facilitated by a compact genome, a large set of well-characterized ecotypes and the availability of a large set of molecular biology tools[25,26,27]. These genes include biosynthetic enzymes, regulatory transcription factors and transporters [19,20,28] (Fig 1). For example, the locus *GS-ELONG* controls variation in the side-chain length of aliphatic glucosinolates, and maps to the *MAM* genes, encoding enzymes involved in chain elongation [29]. The *GS-OX* locus controls the ratio between methylthioalkyl to methylsulfinylalkyl GLS [30], comprising five monooxygenase isogenes which mediate conversion of methylthioalkyl GLS. Two loci control the presence of hydroxylated aliphatic GLS. The *AOP* locus encodes dioxygenases which convert methylthioalkyl GLS to either hydroxyalkyl GLS (*AOP3*), or alkenyl GLS (*AOP2*) [31]. The *GS-OH* locus encodes another dioxygenase, which converts alkenyl GLS to 2-hydroxyalkenyl GLS [32]. In addition, several studies describe transcription factors that regulate GLS biosynthesis in Arabidopsis [5]. For instance, *MYB28*, *MYB29* and *MYB76* were all identified as positive regulators for the production of aliphatic GLS of different chain lengths, which reciprocally trans activate each other, while *MYB29* also plays a role in jasmonic acid-induced aliphatic GLS biosynthesis [33,34,35]. *MYB51* and *MYB34* were identified in *A*. *thaliana* as regulators of indolic GLS [36]. Recently, GLS transporters have been identified, that import GLS from the apoplastic space to the symplast [27,37]. More loci controlling GLS variation, GLS storage and GLS breakdown in Arabidopsis have been reviewed [38].

Genetic research of non-model species such as turnip benefits from the pioneering work in Arabidopsis, since biochemical pathways in *B*. *rapa* are not fundamentally different from those in Arabidopsis [39]. However, the tuber tissue from turnips is absent from the architecture of Arabidopsis, and acts both as a sink during vegetative development, and as a source during generative development of the plant. In view of this important physiological role, the identification of genetic loci determining the specific GLS composition in turnip tuber tissue is relevant. While the Arabidopsis genome is compact, the *B rapa* genome is the result of a genome triplication from a common ancestor of *B*. *oleracea* and *B*. *rapa* [40]. Gene loss, due to genome fractionation after the triplication event, has led to the disappearance of gene paralogs [41,42]. Gene loss is not a random process, as some gene classes are more strongly retained compared to others [41,43]. In two genome wide studies orthologues of *A*. *thaliana* GLS genes and their genetic positions in *B*. *rapa* were identified showing that many paralogues had been retained [42,44]. Different paralogues likely have different roles depending on plant genotype, age and organs, yet this has barely been studied. For example, [45] showed that the three *MYB28* paralogues in *B*. *rapa*, each of them encoding a functional protein, have clearly distinct expression patterns, with one copy expressed in none of the tissues tested.

In the present work, the accumulation of 11 GLS forms in different tissues and developmental stages of two turnip accessions has been investigated, revealing the dynamics of GLS accumulation during the lifecycle of *B*. *rapa*. This information was complemented by a gene expression analysis, probing expression of a range of *B*. *rapa* paralogues of genes that have been identified as relevant for GLS biosynthesis, regulation and transport in Arabidopsis. Subsequently it is discussed in how far this information can be deployed to preselect genetic loci and markers for breeding for specific GLS content and composition in turnip.

## Material & methods

### Plant material

Both FT-004 (Lange Gele Dortfelder, CGN06678, originating from Denmark) and FT-086 (CGN0722, originating from Pakistan) were kindly provided by The Centre for Genetic Resources, the Netherlands (http://www.cgn.wur.nl/UK/). These accessions had previously been shown to have contrasting GLS profiles in both tuber and leaf tissues [4,17] while developing synchronously. The growth experiment was organized as a completely randomized block design with three blocks in a single greenhouse compartment at Unifarm (Wageningen University and Research), to minimise environmental differences (for day and night temperature profile, see S1 Table). The seeds were sown on the 7th of July 2010 and immediately after emergence transplanted to Jiffy pots (July the 12^th^). Three weeks after sowing, 90 plantlets of each accession were transplanted to larger pots (diameter 15 cm) and 30 plants of each accession were randomly distributed over each block. During winter the compartment was kept frost free, so that the low temperatures induced flowering. Both accessions started flowering April 2011.

Four crosses were made between FT-004 (mother) and FT-086 (father). About 10 seeds from each cross were germinated (March 1^st^ 2019) and for each cross 3 representative turnip tubers were harvested in April 10^th^ 2019.

### Collection of tissues for glucosinolate profiling and gene expression analysis

At seven time points during the plant life cycle, different tissues were harvested (Table 1). After tissue collection plants were removed, so each plant was only harvested once. For each harvest, tissue samples were taken from three plants per block, and pooled per accession per block into a corning tube, and then immediately frozen in liquid nitrogen and stored at minus 80°C. So from each tissue, three biological replicates (blocks) were analysed separately. From the turnip tuber we collected two tissues, the outer tissue and the inner core. For the outer turnip tissue we basically took slices from the peel, for the inner turnip tuber tissue, a 1 cm diameter cork borer was used to collect a horizontal cross section through the entire tuber for each plant. Each sample was immediately frozen in liquid nitrogen.

**Table 1:** Harvesting time, properties and analyses of materials from FT-004 and FT-086.

Time point	Date	Tissue harvested									
		seedling	young leaves	mature leaves	tuber core	tuber surface	stem	flower	seed	diam turnip (cm)	rosette leaf size (cm)
**T1**	**26-7-2010**	**g,e**									**5–10**
**T2**	**16-8-2010**		**g,e**	**g**	**g,e**	**g**				**1–2**	**40**
**T3**	**30-8-2010**		**g**	**g**	**g,e**	**g**				**3–4**	**60**
**T4**	**16-9-2010**		**g**	**g,e**	**g**	**g**				**5–7**	**60**
**T5**	**29-9-2010**		**g**	**g**	**g**	**g**				**8–10**	**60**
**T6**	**5-4-2011**		**g,e**	**g,e**	**g,e**	**g**	**g,e**	**g,e**		**12–15**	**bolting**
**T7**	**21-6-2011**								**g,e**	**hollow**	**senescent**

g: glucosinolate profile generated; e: gene expression profiled

FT-086, FT-004 and F1 seeds were planted on March 1^st^ 2019 and turnip material was harvested on April 10^th^ 2019 for GLS analysis.

The harvest dates and the tissues collected are displayed in Table 1. At the latest developmental stage, most tissues had senesced and we only harvested the turnip tuber tissues and seeds.

### Glucosinolate profiling

Samples were ground in liquid nitrogen and freeze-dried. As the current study is predominantly a comparative analysis, and the analysis of intact GLS has previously been shown to be well correlated to desulpho GLS but much less prone to enzyme batch variations, the method described before [4,46]. Intact GLS were determined using HPLC coupled to both UV/Vis and accurate mass detection (LC-PDA-QTOF MS; in short: LC-MS). Samples were extracted from 50 mg freeze-dried powder with 2 mL of 75% methanol and 0.1% formic acid for 15 min by sonication, and then centrifuged for 15 min at 4°C at 1000g. After centrifugation of the crude extracts, the supernatants were filtered using Minisart SRP4 filters (Sartorius, Goettingen) for LC-MS analysis. Each extract was injected (5 μL) into an Alliance 2795 HT instrument (Waters) and separated on a C18 column (Phenomenex Luna, 2.0 mm × 150 mm, 3 μm particle size). Eluents used were water and 0.1% formic acid (A) and acetonitrile and 0.1% formic acid (B). Compounds were separated using a gradient from 5% B to 35% B in 45 min and then detected firstly by a PDA detector (wavelength 220−600 nm, Waters Co.) and secondly by a QTOF Ultima mass spectrometer (Waters Co.) with negative electrospray ionization (m/z 80−1500). Eleven different GLS peaks were identified based on their specific accurate masses and retention times [4] (S2 Table). The relative levels of GLS were determined by integrating the chromatographic peak areas of their molecular ions (within 5 ppm mass deviation) using the QuanLynx module of MassLynx LC-MS software (Waters Co.).

### Anova analysis of GLS data

Effects of tissue, genotype, time and all interactions were analysed by linear modelling / ANOVA in R, version 3.4.0 (https://www.R-project.org/).

### Gene expression profiling

We profiled a subset of genes involved in GLS biosynthesis, regulation of biosynthesis and GLS transport, over a subset of the samples of both turnip genotypes. Selection of samples was based on assessment of GLS profiles, with selection of samples (tissues and developmental stages) when GLS profiles displayed major changes (see S4 Table). In S3 Table the selected *B*. *rapa* gene orthologues and primers used for their amplification in the qrtPCR analysis are listed.

Total RNA was extracted from the stored frozen tissues using the TRIZOL reagent (Invitrogen). Genomic DNA contaminations were effectively removed using RNase-free DNase I treatment (Invitrogen, Carlsbad, CA, USA) according to manufacturer’s instructions. cDNA synthesis was performed with the iScriptTM kit (BIO-RAD) according to supplier’s instructions. A final cDNA concentration of 40ng/ul sample was obtained by dilution of the cDNA with RNA free MQ water, which was used for real-time RT-PCR. qRT–PCR reactions were performed in a 96 position carousel (Light Cycler) with the Light Cycler-RNA amplification kit SYBR Green I (Roche, Mannheim, Germany). A final volume of 10ul per reaction contained 1 μL of cDNA; 5ul of SYBR Green Supermix; 3.4 μL of RNA free MQ water; 0.3 μL of Forward Primer at 10uM and 0.3 μL Reverse Primer at 10uM. The thermal cycling consisted of 95°C for 2 min and 40 cycles of 95°C for 20s, 55°C for 20s and 72°C for 20s. All the cycle threshold (Ct) values from one gene were determined at the same threshold fluorescence value of 0.2. Three technical replications were used for each time point in the experiment. ACTIN (ACT) was used as reference gene in all expression studies. It displayed the most constant expression level relative to total RNA content among three tested genes, including tubulin and elongation factor (not shown). For seed samples, high variability was observed for all tested reference genes. Analysis of the expression data was performed using the Rotor-gene 6 ver. 6.1 software (Applied Biosystems). Quantitative PCR data above Ct value 32 were considered as absence of expression.

### Multivariate analysis

After range-scaling transformation [47], both relative levels of GLS and gene expression were imported into GeneMaths XT version 2.12, build 2 (Applied Maths, Sint-Martens-Latem, Belgium) for Principal Components Analysis (PCA) in order to get insight into differences in GLS levels and gene expression between the tissues and developmental stages of the two contrasting accessions. The PCA’s were based upon accession-tissue-time point combinations of the samples. For the PCA on the GLS data, the average of the three biological replicates were taken. In the case of gene expression data the means of the relative gene expression (−𝛥𝐶𝑡) of two biological replicates were used.

## Results

### GLS analysis

Two accessions of turnip, FT-004 from Denmark and FT-086 from Pakistan, were selected from a previous analysis [4], since their development proceeded synchronously, with regard to turnip tuber formation and flowering. On the other hand, GLS content of their tubers and leaves differed strongly. Plants of both accessions were raised in a large number of replicates in a complete block design under identical conditions, and material from seedlings, juvenile and adult plants was collected including young leaves, mature leaves, tuber core, tuber surface, stem, flowers and seeds at different time points (Table 1). Intact GLS were extracted from these materials and analysed by LC-MS. Eleven different GLS were identified [4], and their relative levels in the different samples were compared. A list of GLS and the specific masses used for quantification are presented in S2 Table while the relative levels of the GLS are presented in S4 Table.

### Global variation in GLS content

**Fig 2:**
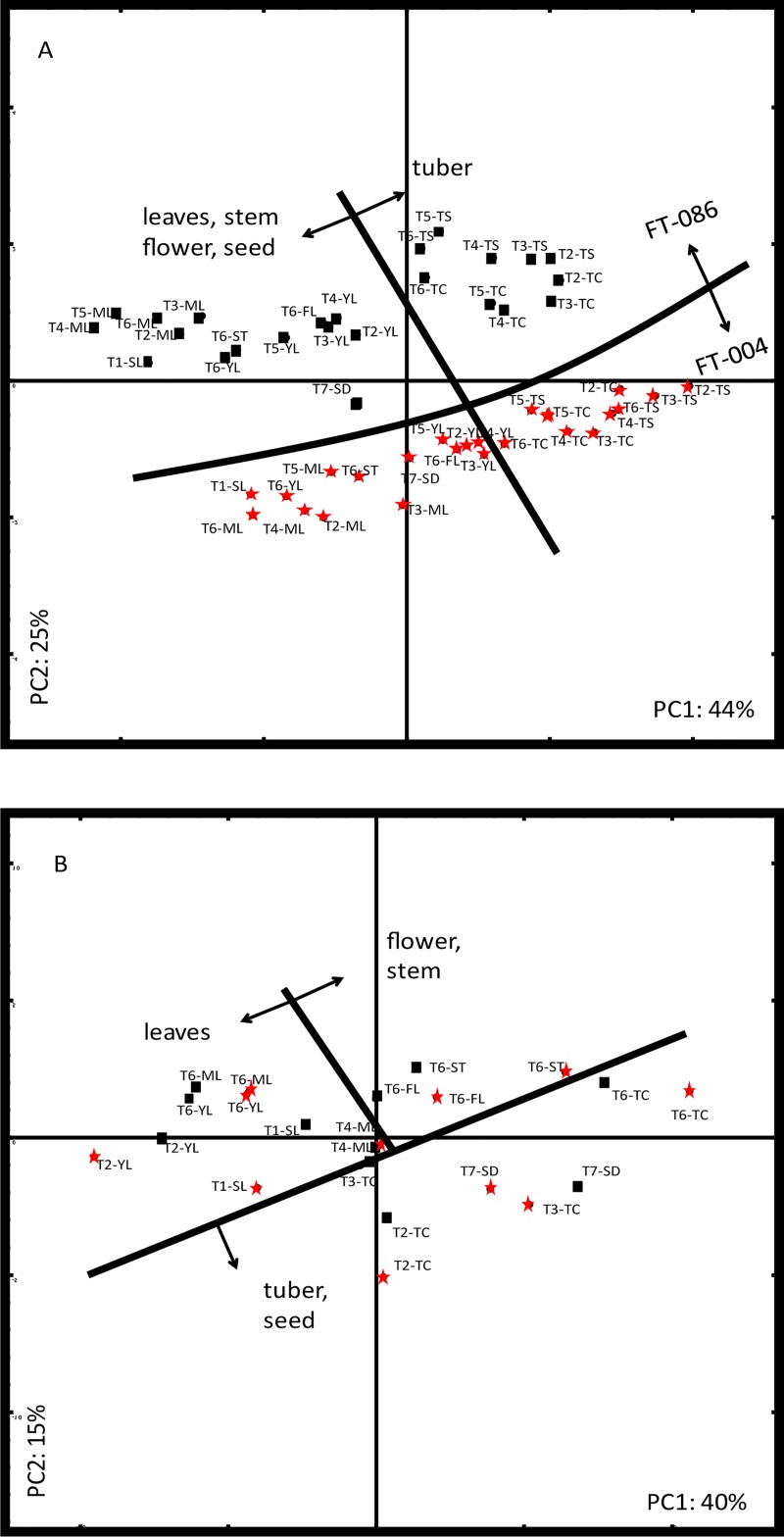
Principle component analysis of GLS data (A) and gene expression data (B) of investigated turnip tissues from two accessions at different timepoints. Average values of three biological replicates were used for each datapoint. Accessions: black squares: FT-086; red stars: FT-004. Tissues: FL: flower; ML: mature leaves; SD: seed; SL: seedling; ST: stem; TC: tuber core; TS: tuber surface; YL: young leaves. Timepoints: T1: 20 days after germinating; T2: 40 days; T3: 54 days; T4: 71 days; T5: 84 days; T6: 272 days; T7: 349 days.

Principle component analysis (PCA) of GLS data was performed to visualize the observed differences in samples based on their differential GLS profiles (Fig 2A). The first principal component (PC), explaining 44% of the total variation, mostly corresponds to the different tissues. In particular, the tuber samples, from both skin and core, separated from all the above-ground tissues, including seedlings, leaves, flowers, seeds and stems. The second PC, which explained 25% of the total variation, clearly separates the two turnip accessions. These differences between tissues and accessions are due to differences in specific GLS, as is clear from the PCA-loading plots visualizing the distribution of GLS species across the various turnip samples (S1A Fig). We tested whether there were significant effects of genotype, tissue and time-point and their interactions on the content of individual GLS (S5 Table). For both time and tissue-type, significant (p<0.05) effects were observed for all GLS. There was also a significant genotype effect for all GLS, except for BRAS and NAS. Most interactions were also significant, however for several GLS there was no interaction between time and genotype. GLS were addressed in more detail to reveal which GLS are at the basis of the global differences observed in the PCA (Fig 2).

### Genotype specificity for GLS

**Fig 3:**
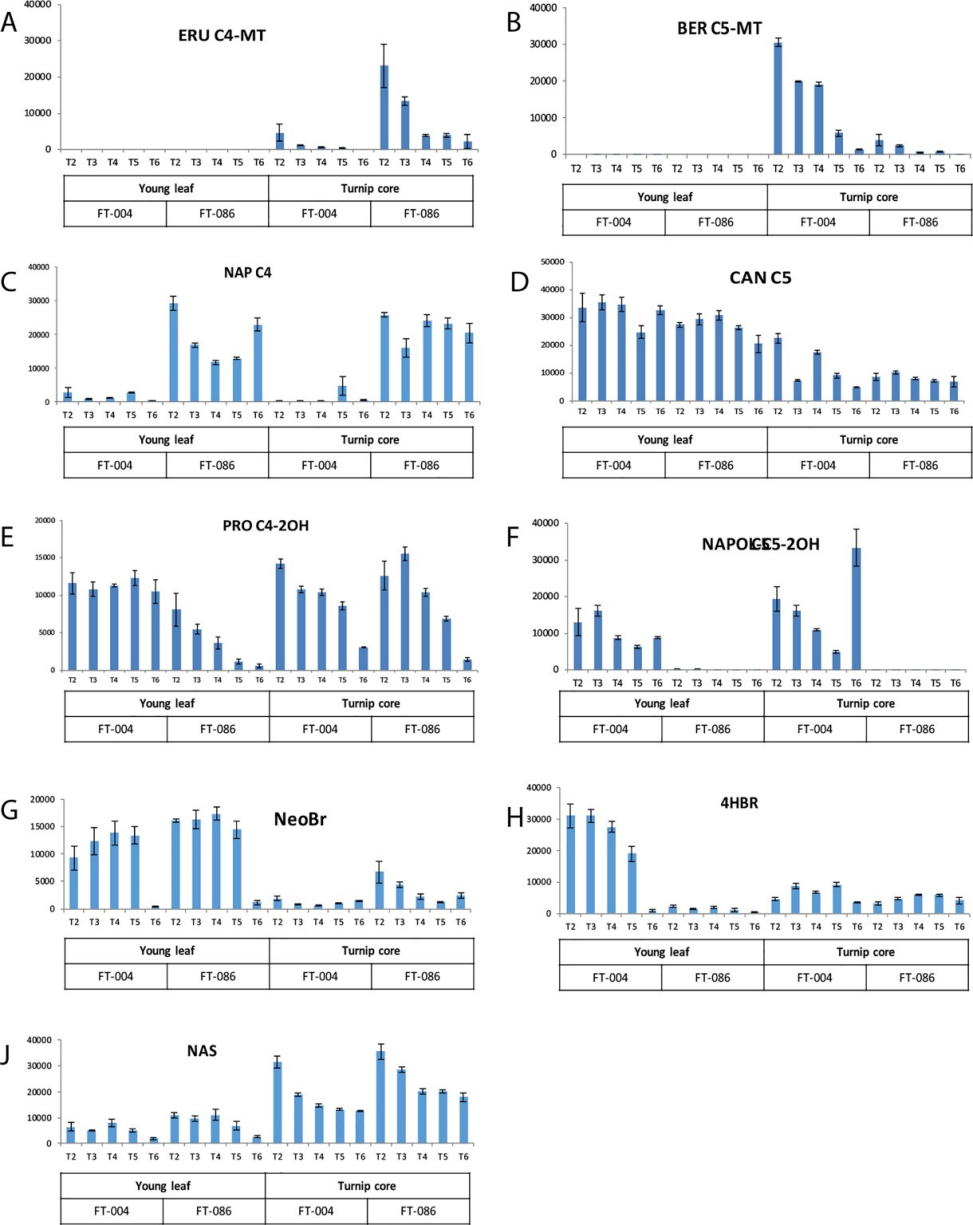
Relative quantity of individual GLS (A-J) in turnip tissues in FT-004 and FT-086 accessions. The Y-axis shows the peak surface area measured in LC-MS for the indicated compound. For timepoints see legend Fig 2. Error bars indicate standard deviation (n = 3).

Accessions FT-004 and FT-086 differed in many GLS from each other (Fig 3). Glucoberteroin (BER; Fig 3B) was detected at much higher levels in tubers of FT-004 than of FT-086. FT-004 had also a much higher content of gluconapoliferin (NAPOL; Fig 3F), both in tubers and in young leaves. Similarly, the amount of 4-hydroxybrassicin (4-HBR; Fig 3H) was generally much higher in FT-004 tissues than in FT-086. On the other hand, glucoerucin (ERU; Fig 3A) levels were higher in FT-086; also gluconapin (NAP; Fig 3C) was higher in FT-086, although in young leaf material this was much less pronounced than in tuber material and in mature leaf material.

### Tissue specificity for GLS

Turnip tubers display a more diverse GLS complement than turnip leaves. For example, ERU and BER occurred in well-detectable levels in tubers, but were hardly detectable in leaves, stem, flower or seed (Fig 3A and 3B, S4 Table). Also NAS was highest in tubers, compared to the other tissues. Gluconeobrassicin (NeoBr; Fig 3G), on the other hand, was more abundant in leaves. It may be of interest to note that tissues like flowers, stems and seeds, which only occurred after winter had passed, generally compare well to leaf material, and do not contain exceptionally high levels of GLS (S4 Table).

### GLS during development of the plant

Few GLS decreased during plant development. The relative content of BER and ERU in turnip tubers each were reduced during turnip maturation (T2-T5) in either genotype (Fig 3D, 3C and 3H). Interestingly, concentrations of the indole GLS such as NeoBr and 4HBr (Fig 3G and 3H) in leaf material dropped strongly in the transition from vegetative stages (T1-T5) to flowering stage (T6). For other GLS, such as CAN (Fig 3F) no such trends can be observed and levels seem to be rather constant during plant development. Interestingly, PRO did gradually decrease during plant development in leaves of FT086 and did not in FT004 (Fig 3E).

### Aliphatic GLS side chain modification in turnip

With regard to specific modifications on side-chains of aliphatic GLS, we observed that aliphatic GLS with a C5 side chain (BER, CAN, NAPOL) were generally more predominant in FT-004, while aliphatic GLS with a C4 side chain (ERU, NAP) were more dominant in FT-086. Also, the C5 aliphatic GLS with a 2-hydroxy group on the side chain (NAPOL) was exclusively detectable in FT-004, while its C4 counterpart (PRO) was present in comparable amounts in both genotypes. GLS with a methylthio- group terminating the aliphatic side chain (BER, ERU) occurred almost exclusively in turnip tubers, and hardly in above-ground tissues.

### GLS in F1 from a cross between FT-004 and FT-086

GLS were profiled in tubers of the two parental genotypes and their F1 plants at 40 days after sowing (S4 Table). The F1 resembled the FT-004 parent for the aliphatic GLS, as the relative abundancies of the C5 GLS (BER, CAN and NAPOL) are much higher than those in FT-086. Their abundancies are however somewhat lower than in FT004 tubers, especially for BER and NAPOL (S2 Table).

### Gene expression analysis

**Fig 4:**
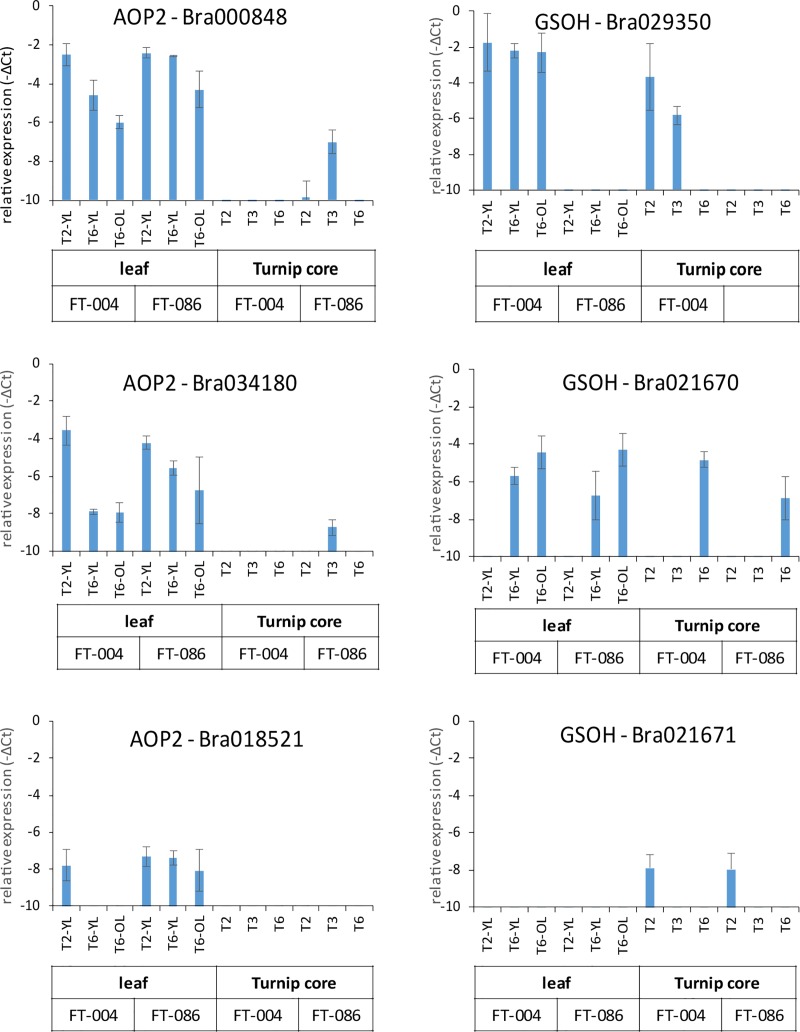
Gene expression analysis of *AOP2* and *GS-OH* paralogues in selected turnip tissues and timepoints in accessions FT-004 and FT-086. Indicated are the expression levels relative to the reference gene (actin) on a logarithmic scale. Error bars indicate standard deviations (n = 3). YL: young leaf; OL: old leaf. For timepoints see legend Fig 2.

Genes (S3 Table) involved in aliphatic, indole and aromatic GLS pathways were selected from a collection of GLS genes identified in the *B*. *rapa* genome [42]. Expression of these genes was tested in a limited number of samples (Fig 4), including seedlings, young leaves, old leaves, turnip core, turnip surface, bolt, flower and seed by quantitative RT-PCR, using paralog-specific primer pairs and actin as a reference gene (S6 Table; S6 Fig).

Principle component analysis (PCA) of gene expression data was performed to visualize the observed differences in gene expression profiles (Fig 2B). The first component, explaining 40% of variation, separates different tissues. In particular, turnip tuber samples were separated from leaf tissue. Samples of seedlings were found amidst the leaf samples. The second component, which explains 15% of the total variation, seems to separate different harvest dates, as in the top quadrants predominantly T6 samples are positioned, while in the lower quadrants, leaves and turnip tubers from T2 and seedlings and seeds are found (S1B and S5 Figs). Clearly, global gene expression did not explain the observed differences in GLS profile. Therefore, a few individual genes were addressed in more detail to possibly explain observed differences in GLS profiles in turnip samples.

#### *GSOH Bra022920* is co-regulated with NAPOL

The relative abundance of the 2-hydroxylated GLS NAPOL in accession FT-004 could relate to a difference in expression of a paralog of Arabidopsis *GS-OH*. In Arabidopsis this enzyme catalyses the 2-hydroxylation of alkenyl-GLS [32]. In the *B*. *rapa* genome, three paralogs of *GS-OH* have been identified: *Bra212670*, *Bra021671* and *Bra022920* [42]. These three paralogs displayed completely different behaviour (Fig 4B, 4D and 4F). Only *Bra022920* is highly expressed in both leaf and turnip of accession FT-004, while its expression is hardly detectable in FT-086, consistently with the NAPOL concentration.

#### *AOP2* paralogs are oppositely regulated with BER and ERU

The absence of the methylthio GLS BER and ERU in above-ground tissues, and their presence in tuber tissue could relate to a difference in *AOP2* expression. *AOP2* in Arabidopsis is known to mediate the conversion of methylthio GLS into alkenyl GLS [31]. All three paralogs of AOP2 (*Bra000848*, *Bra018521* and *Bra034180* [42]) were hardly expressed in tuber tissue, while much higher expressed in leaf tissue (Fig 4A, 4C and 4E). We hypothesize that *AOP2* in leaves metabolizes BER and ERU, resulting in their very low levels in leaf tissue.

#### Differences in GLS chain length and the expression of *MAM* genes

One of the striking differences between accessions FT-004 and FT-086 is the chain-length of their aliphatic GLS. In Arabidopsis, chain-length of GLS is known to be controlled by the *GS-ELONG* locus, where *MAM1*, *MAM2* and *MAM3* control chain length. In the *B*. *rapa* genome, three *MAM1* genes (*Bra013007*, *Bra029355* and *Bra018524*) and four *MAM3* genes (*Bra013009*, *Bra13011*, *Bra029356* and *Bra021947*) have been identified[42]. Only the *MAM3* paralogue *Bra013009* is higher expressed in early stages of FT-004 than in FT-086 and could be responsible for chain elongation from C4 to C5 (S3 Fig).

#### Gene expression suggests that the major sites of biosynthesis of aliphatic GLS are above-ground

A correlation analysis of expressed genes in the *B*. *rapa* genotypes across all tested tissues of both accessions (S4 Fig) revealed strong correlations between *MYB28* (both *Bra012961* and *Bra035929*) and most of the tested aliphatic biosynthetic genes which was not the case for *MYB29* (*Bra005949*). Remarkably, both *MYB28* genes display a pattern where they are well expressed in the leaf materials, but poorly detectable in the tuber tissues.

## Discussion

During the life cycle of a plant, metabolites are needed at different times and for different purposes. For example, the tuber tissue of a turnip plant initially functions as a sink to store nutrients for the plant, while it will function as a source to supply these nutrients when the plant goes into the reproductive stage and starts bolting, flowering and setting seed. Leaves provide photosynthetic capacity, and are replaced continuously by young fresh leaves during the life cycle of the plant; therefore they likely have different requirements for their functioning than turnip tubers. These requirements are at least partly met by their chemical composition. In recent years, changes in chemistry and nutritional status during sink-source transitions have been addressed on the level of sucrose transport (e.g. [48]), but much less on the level of secondary metabolites. However, in addition to differences in physiological roles of leaves and tubers, their chemistry is also under selection pressure to defend the plant to different biotic stresses, such as insects, snails, vertebrate herbivores, fungi and bacteria. Hence, differences in secondary metabolites such as GLS can be expected between the different tissues of turnip plants. Two recent reviews address these issues. Jørgensen et al. [20] present what is known about transport of defence compounds from source to sink, and use GLS as a case study. They discuss especially the roles of GLS transporters in establishing dynamic GLS patterns in Arabidopsis source and sink tissues. Burow and Halkier [19] also use GLS as case study and discuss how Arabidopsis orchestrates synthesis, storage and mobilization to target tissues.

In this work, we studied the composition of GLS of different tissues of *B*. *rapa* ssp. *rapa* (turnip), during the life cycle from seedling to seed. While in Arabidopsis and Brassica species like oil seed crops, the sink is the inflorescence with developing seeds, and no intermittent tuber storage organ is present, turnip is a crop which forms a tuber, which is initially acting as a sink, and later as a source for the developing seeds. Therefore we reasoned that turnip would present an interesting case for studying GLS content in different stages of its lifecycle. Since we have noticed before that GLS composition can differ significantly between turnip genotypes, it is of interest to compare several accessions [4]. Initially, more turnip accessions were grown for this comparison, but were omitted since they appeared to flower at different times, varying between 3 and 14 months after germination. The current analysis was restricted to two specific genotypes with highly synchronous life cycles, but pronounced differences in GLS profiles and geographic origin.

A first conclusion from this work is that GLS profiles are very different between leaf and tuber material. Some GLS were relatively abundant in tubers, and undetectable in turnip leaves. This was particularly true for methylthio group GLS, such as ERU and BER. This difference in GLS likely translates in differences in taste and mouthfeel, but also in resistance to pathogens. Breakdown products of these ERU and BER (the isothiocyanates erucin and berteroin) have a penetrating radish like aroma [49]. Also NAS is much higher in tuber tissue than in leaf tissue. The breakdown product of NAS (benzylisothiocyanate) provides a watercress flavour, and a tingling sensation on the tongue. Interestingly, in a recent study GLS profiles of both leaf and turnip tissue of 16 turnip accessions were analyzed [23]. In this study, GLS profiles between leaves and tubers also differed, with turnip tubers generally having higher amounts of GLSs than leaves, and also different GLS composition. Tubers were particularly rich in the phenylethyl GLS NAS, like in our study, and in progoitrin. However the GLS BER and ERU were not described for turnip tissues, possibly since different genotypes were analyzed.

Interestingly, both erucin and benzylisothiocyanate, have a high nematicidal activity on pathogens such as *Meloidogyne*. Nematodes are particularly a pest for underground tissues [50], which could explain why the turnip specifically produces these nematicidal compounds in the roots. This is exploited by breeding turnips for cover crops that are incorporated as green manures prior to transplanting of vegetables [51].

A second observation that can be made is that a number of GLS in turnip tuber tissues (ERU, BER, NAS) decline during the life cycle. For leaf tissues such decline cannot be observed, and a constant or an increasing GLS content of both aliphatic and indolic GLS is observed (Fig 3). These trends seem to be opposite of those observed in Arabidopsis, where aliphatic GLS in rosette leaves decline during aging of the plant, but indolic GLS increase [52]. Interestingly, at timepoint T6, where the plant is flowering after winter rest, the indolic GLS (BRA, NEO, 4HBRA and 4MBRA) are strongly reduced, in particular in leaf tissue (Fig 3, S4 Table).

Genotype-dependent differences in GLS content can be clearly observed. It is not clear yet what the consequence of these differences between FT-004 and FT-086 (FT-004 tissues high NAPOL and C5 compounds, FT-086 high in NAP and C4 compounds) will be with respect to taste and/or insect resistance. The breakdown products of NAP and NAPOL are both associated with cabbage-like, aromatic pungency, and sulphur-like impressions, which are characteristic for leaves, but very little is known about subtle differences in taste or bio-activity of these compounds.

### Gene expression data

Clearly, there are no global changes in gene expression that are responsible for the differences in GLS. This can be seen when the PCA analyses of GLS compounds and biosynthetic genes are compared (Fig 2). Both PCAs separate the tissues differently. This is especially the case for seeds, that group with leaves for the GLS and with tubers for the biosynthesis genes. One reason why it may be complicated to find direct relations between GLS biosynthesis gene expression profiles and GLS is the role of transport of GLS between the different tissues. Expression profiles of three paralogues of transporter gene *GTR1* and four of *GTR2* were tested, but no clear differences between tissues and genotypes could be observed, so that it is difficult to draw conclusions about the role of the individual transporter paralogues (S6 Table). Latest research revealed that the transporters *GTR1* and *GTR2* import GLS from the apoplastic space to the symplast [27,37], however their role in long distance transport is not yet clear.

For some of the tested biosynthetic genes, indications for the roles of different paralogues could be obtained. For example, *GS-OH Bra022920* is a potential locus for determining the presence or absence of NAPOL in the two genotypes. From three candidate *GS-OH* paralogues, only *Bra22920* showed a strong differential expression between the two genotypes, and is also expressed at significant levels in tissues where NAPOL is present.

Metabolite data suggest that *AOP2* is also a major determinant of GLS composition. *AOP2* paralogues are not expressed in turnip tuber, where, consistently, the AOP2 substrates ERU and BER accumulate. All three *AOP2* loci tested follow this behaviour, and therefore, in spite of differences in expression levels which possibly could point to *Bra000848* as the major important *AOP2* paralogue, the gene expression data do not allow to differentiate the role of different paralogues.

Finally the metabolite data point to a role for the *MAM* locus. C5 NAPOL and BER are hardly present in FT-086. This could point to a difference in *MAM3* activity between the two genotypes. Only one (*Bra013009*) of the seven *MAM* paralogues observed in the *B*. *rapa* genome follows this behaviour. When inspecting the protein encoded by *Bra013009* in the *B*. *rapa* genome, a truncated polypeptide is observed, which lacks a large part of the N-terminus (S5 Fig). Differences in elongation capacities, which do exist for Arabidopsis *MAM* genes, are very difficult to infer from the *MAM* protein primary structures. In fact, *MAM1* and *MAM2*, which differ in the chain length of the aliphatic GLS they produce, have >95% identity in amino acids [53]. The *MAM*-containing loci in Arabidopsis display more of such truncated proteins, and the *MAM* locus is prone to gene rearrangements, as has been observed in Arabidopsis [53]. The organization of the *MAM* locus and *MAM* paralogues is not known in the two accessions used for this study, however from resequencing studies it became clear that structural variations are common when comparing three *B*. *rapa* genotypes [46]. In a recent paper genome sequences of ten *B*. *oleracea* varieties representing the different morphotypes were used to construct a so called Pan genome [54], which illustrated that nearly 20% of genes are affected by presence/absence variation. From the genes displaying presence/absence variation several were annotated with functions related to major agronomic traits, among others GLS metabolism and vitamin biosynthesis. As *B*. *oleracea* and *B*. *rapa* have similar ancestry and domestication histories, very likely presence absence variation is also a major factor in *B*. *rapa* [55]. To identify the genetic regulation of chain length variation in aliphatic GLS, a genetic analysis of a progeny from a cross between FT-086 (C4) and FT-004 (C5) with similar developmental timing could identify Quantitative Trait Loci involved in chain length differences. Their synchronized developmental timing of the two parents allows to focus on genetic differences, rather than on differences depending on environmental and/or developmental changes. Turnips of F1 plants from crosses between FT-086 and FT-004 showed aliphatic GLS profiles that implied co-dominant inheritance, as the relative abundance was intermediate between that of FT-004 and FT-086. Subsequent genomic DNA sequence analysis of the identified loci in both *B*. *rapa* turnip genotypes would be needed to unravel the genetic base of chain length differences in Turnip GLS. Until this has been done, it will remain unclear which MAM paralog or paralogs are responsible for the observed differences in chain length between the two accessions.

## Conclusion

In this paper, a GLS analysis is presented which aims to provide insight in the chemical changes which accompany development of turnip tissues that function as sinks and sources for the plant. It differs from earlier studies, which focus on single tissues (either leaves or turnip tubers) and or single timepoints. It becomes clear that there are large chemical differences between tissues, between developmental-stages and between genotypes. Clearly these differences will play a role in the eco-physiology of the turnip, given the reported involvement of GLS in plant defence.

To provide mechanistic explanations for the observed changes, more profound analyses are needed, both providing high quality genomic information for the studied accessions, and more comprehensive gene expression analyses using RNA sequencing. The RT-PCR-based gene-expression analysis as presented here already provides putative explanations for the observed differences in profiles. For a true understanding, the role of transport of GLS needs to be further elucidated, which will require more fundamental knowledge. Lastly, to understand the genetic control of GLS profiles of turnip materials for human consumption, such as leaves and tubers, tissue-focussed screening methods should be defined. To develop molecular markers associated with specific GLS profiles for breeding turnips with tailored GLS content will require genomics analyses and analysis of progeny. The results presented here should provide a solid basis for this.

## Supporting information

S1 Table Temperature profile in the greenhouse during turnip cultivation. (XLSX)

S2 Table GLS found in turnip tissues, with chromatographic information and masses used for quantification. (DOCX)

S3 Table Genes and oligonucleotides used for qRT-PCR analysis. (XLSX)

S4 Table Relative quantities of GLS in different analysed turnip tissues. (XLSX)

S5 Table P-values of 11 GLS for the effects of time, tissue and genotype, and the interactions between time, tissue and genotype. (CSV)

S6 Table Relative gene expression of selected genes in turnip tissues, expressed as–ΔCt. (XLSX)

S1 Fig Loading plot of PCA plots for glucosinolates (S1A) and gene expression (S1B). (PPTX)

S2 Fig F1 from cross between FT-004 and FT-086. (PPTX)

S3 Fig Gene expression of *MAM* paralogues. (PPTX)

S4 Fig Correlation matrix for expression of gene paralogues involved in aliphatic GLS biosynthesis in turnip. (PPTX)

S5 Fig Alignment of amino-acid sequences of *MAM* genes from Arabidopsis and their paralogues in *B*. *rapa*. (PPTX)

S6 Fig Heatmap of relative gene expression values in different analyzed tissues, timepoints and genotypes. (PPTX)
