# Supplementary material for: Glucosinolate variability between turnip organs during development
Source: PLoS One. 2019 Jun 6;14(6):e0217862. doi: 10.1371/journal.pone.0217862 (PMC6553741; doi:10.1371/journal.pone.0217862)
Supplement: S2 Table — (DOCX) [file pone.0217862.s002.docx]

**Supplemental table S2.** Chromatographic and mass information for GLS from turnip.

| Trivial name | Abbr. | Side chain | Side-chain (short) | Group | Mass for intact GLS  [M-H]^-^ | Observed RT for intact  GLS (min) |
| --- | --- | --- | --- | --- | --- | --- |
| Progoitrin | PRO | (2*R*)-2-Hydroxy-3-butenyl | 4OHB | Aliphatic | 388.0378 | 3.4 |
| Gluconapoleiferin | NAPOL | 2-Hydroxy-4-pentenyl | 5OHP | Aliphatic | 402.0534 | 5.1 |
| Gluconapin | NAP | 3-Butenyl | - | Aliphatic | 372.0428 | 6.1 |
| 4-Hydroxyglucobrassicin | 4HBRA | 4-Hydroxy-3-indolylmethyl | 4OH-I3M | Indolyl | 463.0487 | 8.1 |
| Glucobrassicanapin | CAN | 4-Pentenyl | - | Aliphatic | 386.0585 | 11.3 |
| Glucoerucin | ERU | 4-Methylthiobutyl | 4MTB | Aliphatic | 420.0462 | 12.7 |
| Glucobrassicin | BRA | 3-Indolylmethyl | I3M | Indolyl | 447.0537 | 15.0 |
| Gluconasturtiin | NAS | 2-Phenethyl | - | Aromatic | 422.0585 | 18.0 |
| Glucoberteroin | BER | 5-Methylthiopentyl | 5MTP | Aliphatic | 434.0619 | 18.4 |
| 4-Methoxyglucobrassicin | 4MBRA | 4-Methoxy-3-indolylmethyl | 4MO-I3M | Indolyl | 477.0643 | 20.5 |
| Neoglucobrassicin | NBRA | *N*-Methoxy-3-indolylmethyl | 1MO-I3M | Indolyl | 477.0643 | 25.8 |
