# Supplementary material for: Glucosinolate variability between turnip organs during development
Source: PLoS One. 2019 Jun 6;14(6):e0217862. doi: 10.1371/journal.pone.0217862 (PMC6553741; doi:10.1371/journal.pone.0217862)
Supplement: S1 Fig — (PPTX) [file pone.0217862.s007.pptx]

## Slide 1
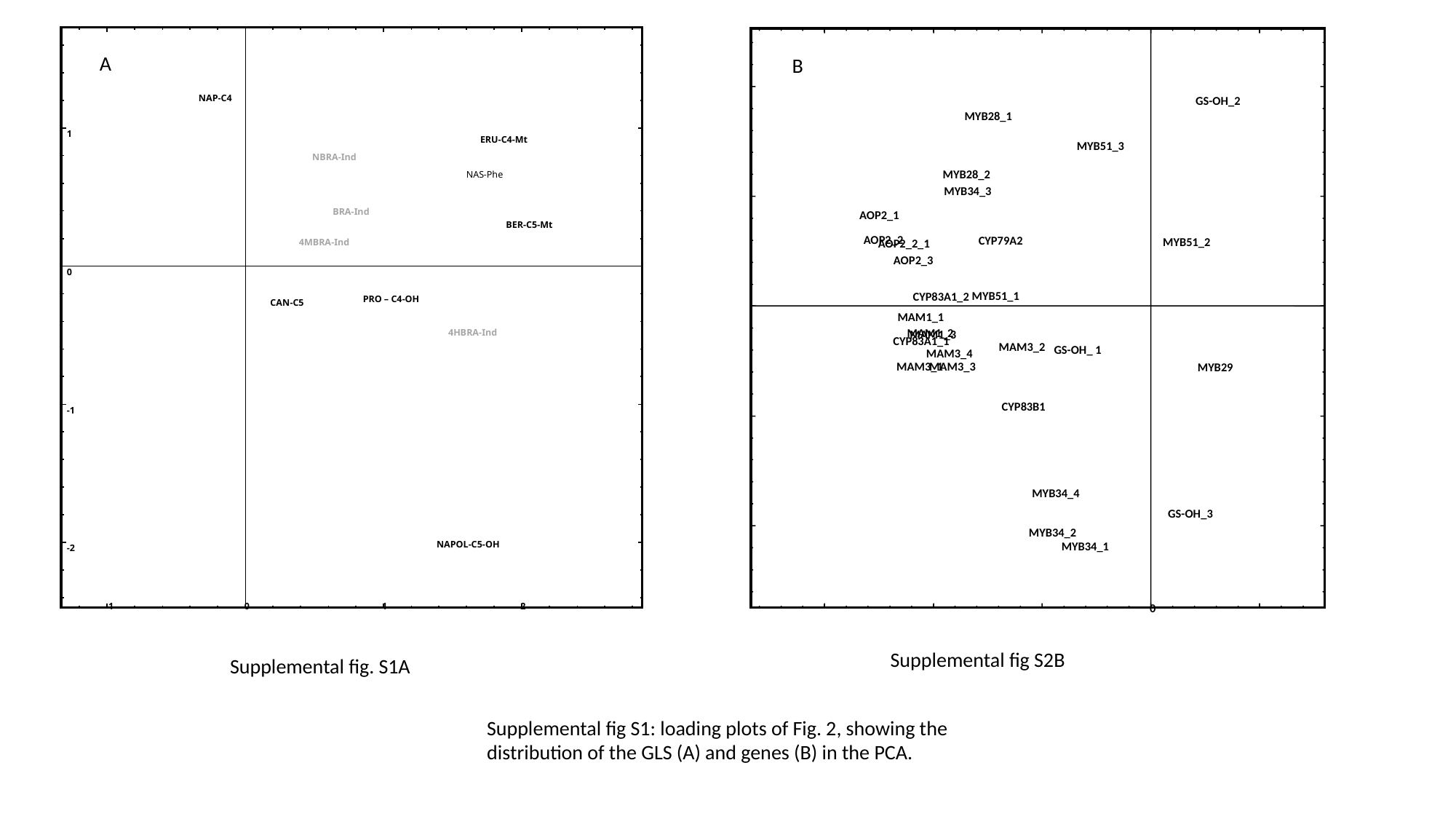

NAP-C4
1
ERU-C4-Mt
NBRA-Ind
NAS-Phe
BRA-Ind
BER-C5-Mt
4MBRA-Ind
0
PRO – C4-OH
CAN-C5
4HBRA-Ind
-1
NAPOL-C5-OH
-2
-1
0
1
2
GS-OH_2
MYB28_1
MYB51_3
MYB28_2
MYB34_3
AOP2_1
AOP2_2
CYP79A2
MYB51_2
AOP2_2_1
AOP2_3
MYB51_1
CYP83A1_2
MAM1_1
MAM1_2
MAM1_3
CYP83A1_1
MAM3_2
GS-OH_ 1
MAM3_4
MAM3_1
MAM3_3
MYB29
CYP83B1
MYB34_4
GS-OH_3
MYB34_2
MYB34_1
0
A
B
Supplemental fig S2B
Supplemental fig. S1A
Supplemental fig S1: loading plots of Fig. 2, showing the distribution of the GLS (A) and genes (B) in the PCA.
