# Supplementary material for: Glucosinolate variability between turnip organs during development
Source: PLoS One. 2019 Jun 6;14(6):e0217862. doi: 10.1371/journal.pone.0217862 (PMC6553741; doi:10.1371/journal.pone.0217862)
Supplement: S2 Fig — (PPTX) [file pone.0217862.s008.pptx]

## Slide 1
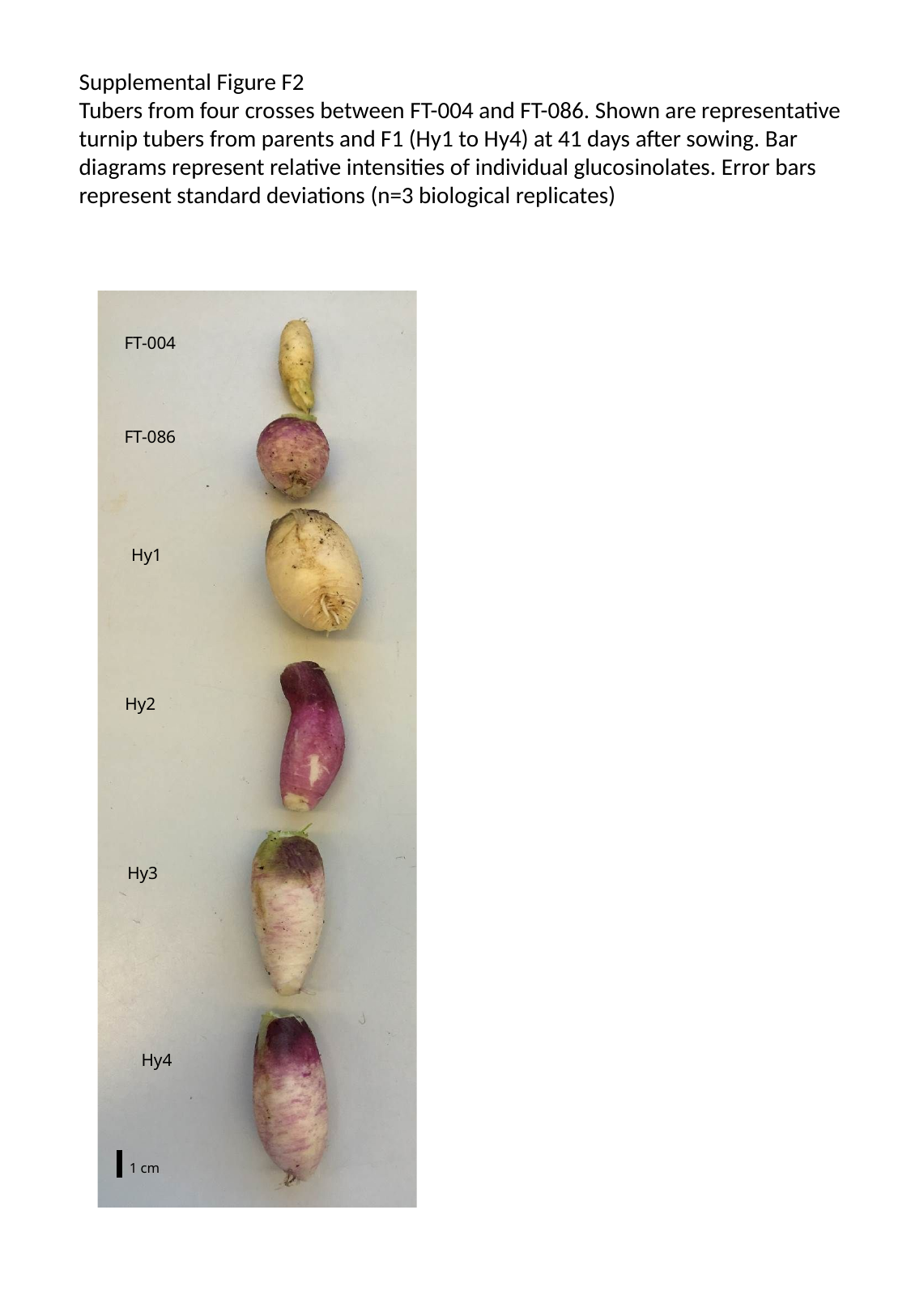

Supplemental Figure F2
Tubers from four crosses between FT-004 and FT-086. Shown are representative turnip tubers from parents and F1 (Hy1 to Hy4) at 41 days after sowing. Bar diagrams represent relative intensities of individual glucosinolates. Error bars represent standard deviations (n=3 biological replicates)
FT-004
FT-086
Hy1
Hy2
Hy3
Hy4
1 cm

## Slide 2
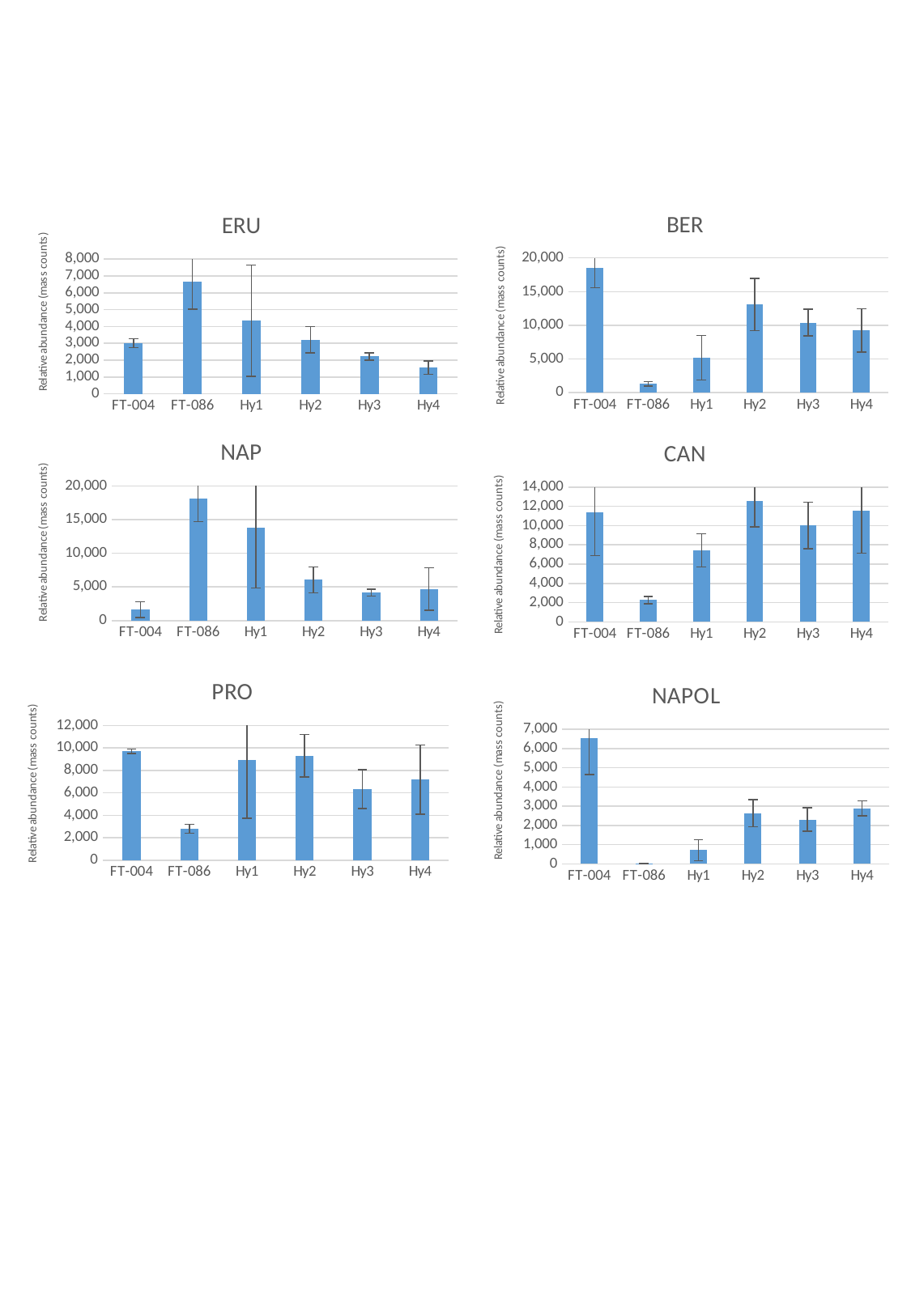

### Chart:
| Category | BER |
|---|---|
| FT-004 | 18550.316766666667 |
| FT-086 | 1294.9183 |
| Hy1 | 5172.267333333333 |
| Hy2 | 13084.759966666667 |
| Hy3 | 10390.823066666668 |
| Hy4 | 9235.4651 |
### Chart:
| Category | ERU |
|---|---|
| FT-004 | 3008.828466666667 |
| FT-086 | 6668.613433333334 |
| Hy1 | 4337.507466666666 |
| Hy2 | 3211.4789333333333 |
| Hy3 | 2219.1729 |
| Hy4 | 1563.6420666666666 |
### Chart:
| Category | NAP |
|---|---|
| FT-004 | 1623.7488666666666 |
| FT-086 | 18104.648033333335 |
| Hy1 | 13749.4038 |
| Hy2 | 6049.3649 |
| Hy3 | 4164.895133333333 |
| Hy4 | 4694.126266666666 |
### Chart:
| Category | CAN |
|---|---|
| FT-004 | 11421.028 |
| FT-086 | 2258.866133333333 |
| Hy1 | 7434.34 |
| Hy2 | 12540.874 |
| Hy3 | 10020.801533333333 |
| Hy4 | 11565.617633333333 |
### Chart:
| Category | PRO |
|---|---|
| FT-004 | 9693.478133333332 |
| FT-086 | 2783.8672666666666 |
| Hy1 | 8955.199066666668 |
| Hy2 | 9293.618033333332 |
| Hy3 | 6328.837966666666 |
| Hy4 | 7175.821 |
### Chart:
| Category | NAPOL |
|---|---|
| FT-004 | 6519.684666666666 |
| FT-086 | 24.5897 |
| Hy1 | 713.1753333333334 |
| Hy2 | 2630.431866666667 |
| Hy3 | 2298.396533333333 |
| Hy4 | 2889.8131 |

## Slide 3
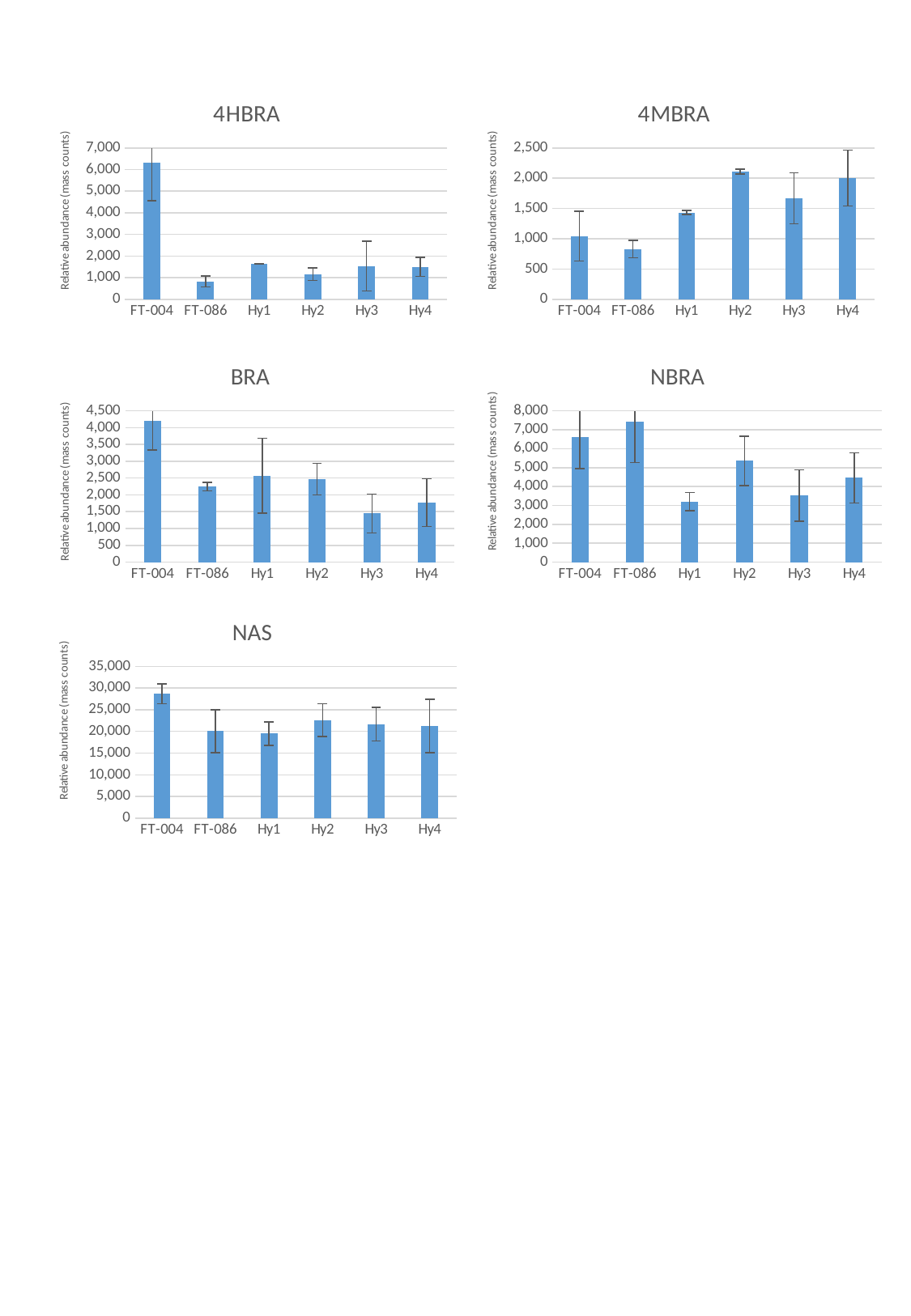

### Chart:
| Category | 4HBRA |
|---|---|
| FT-004 | 6308.1458 |
| FT-086 | 829.2237 |
| Hy1 | 1637.4649 |
| Hy2 | 1157.9286333333334 |
| Hy3 | 1533.6912 |
| Hy4 | 1498.9989333333333 |
### Chart:
| Category | 4MBRA |
|---|---|
| FT-004 | 1043.2684 |
| FT-086 | 829.0423 |
| Hy1 | 1432.7245 |
| Hy2 | 2108.8762333333334 |
| Hy3 | 1667.9526333333333 |
| Hy4 | 2001.3951333333332 |
### Chart:
| Category | BRA |
|---|---|
| FT-004 | 4193.4995 |
| FT-086 | 2245.2026333333333 |
| Hy1 | 2571.8638666666666 |
| Hy2 | 2470.6065 |
| Hy3 | 1448.2422 |
| Hy4 | 1767.2975333333331 |
### Chart:
| Category | NBRA |
|---|---|
| FT-004 | 6605.445133333334 |
| FT-086 | 7440.2663 |
| Hy1 | 3200.9546 |
| Hy2 | 5354.244566666666 |
| Hy3 | 3512.6458666666663 |
| Hy4 | 4452.8365 |
### Chart:
| Category | NAS |
|---|---|
| FT-004 | 28671.80316666667 |
| FT-086 | 20044.903333333335 |
| Hy1 | 19485.134166666667 |
| Hy2 | 22603.965633333333 |
| Hy3 | 21703.130066666665 |
| Hy4 | 21259.421333333335 |
