# Supplementary material for: Glucosinolate variability between turnip organs during development
Source: PLoS One. 2019 Jun 6;14(6):e0217862. doi: 10.1371/journal.pone.0217862 (PMC6553741; doi:10.1371/journal.pone.0217862)
Supplement: S3 Fig — (PPTX) [file pone.0217862.s009.pptx]

## Slide 1
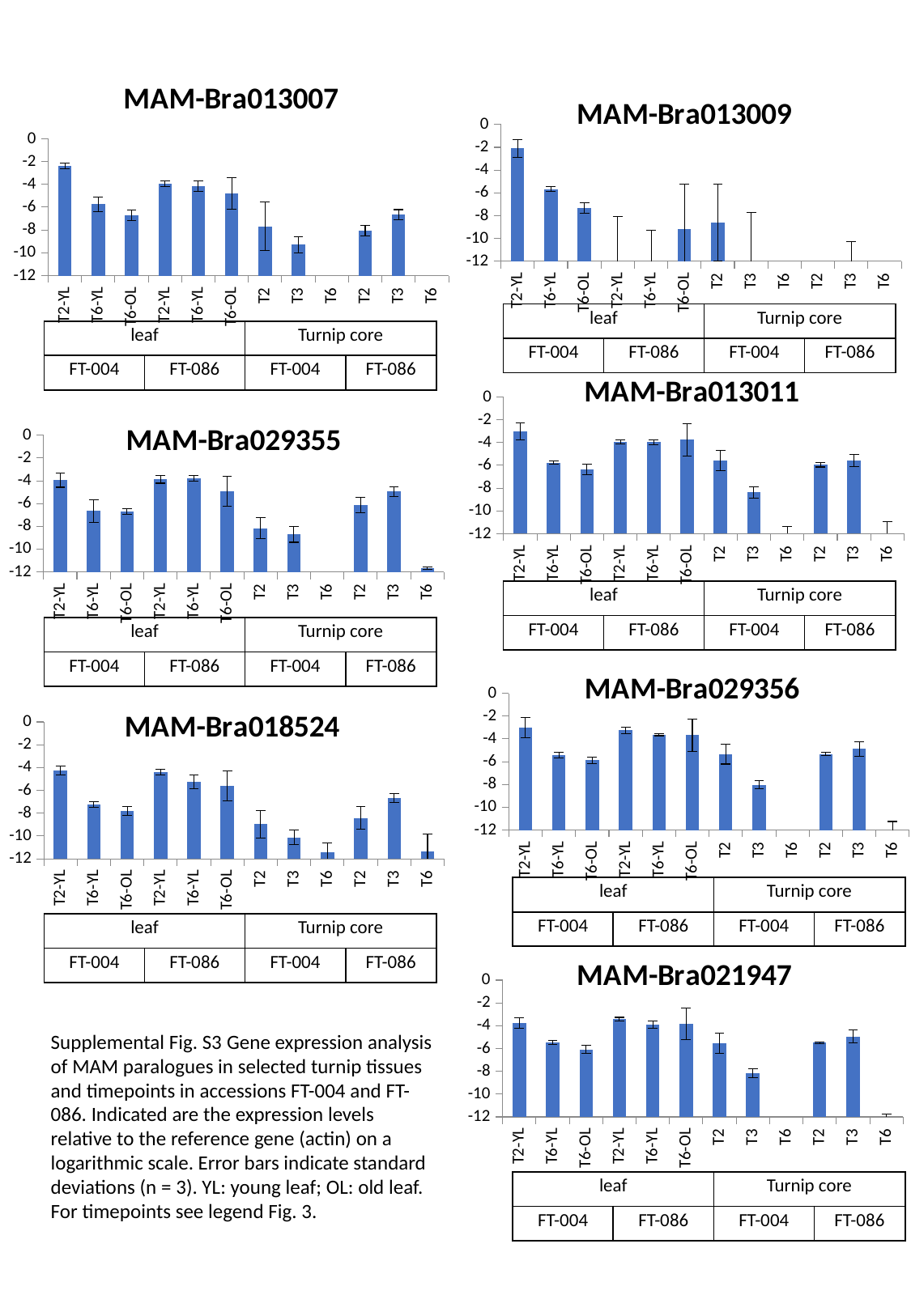

### Chart: MAM-Bra013009
| Category | Mam3-pr-51 |
|---|---|
| T2-YL | -2.0933333333333324 |
| T6-YL | -5.683333333333334 |
| T6-OL | -7.303333333333334 |
| T2-YL | -12.136666666666665 |
| T6-YL | -12.229999999999999 |
| T6-OL | -9.186666666666666 |
| T2 | -8.61 |
| T3 | -12.856666666666667 |
| T6 | -15.053333333333333 |
| T2 | -17.400000000000002 |
| T3 | -14.293333333333331 |
| T6 | -16.743333333333336 |
### Chart: MAM-Bra013007
| Category | Mam1-pr 46 |
|---|---|
| T2-YL | -2.35 |
| T6-YL | -5.753333333333334 |
| T6-OL | -6.7200000000000015 |
| T2-YL | -3.943333333333335 |
| T6-YL | -4.136666666666667 |
| T6-OL | -4.803333333333332 |
| T2 | -7.673333333333333 |
| T3 | -9.303333333333333 |
| T6 | -16.09 |
| T2 | -8.036666666666667 |
| T3 | -6.656666666666667 |
| T6 | -14.116666666666669 || leaf | | Turnip core | |
| --- | --- | --- | --- |
| FT-004 | FT-086 | FT-004 | FT-086 |
| leaf | | Turnip core | |
| --- | --- | --- | --- |
| FT-004 | FT-086 | FT-004 | FT-086 |
### Chart: MAM-Bra013011
| Category | mam3-pr53 |
|---|---|
| T2-YL | -3.0333333333333337 |
| T6-YL | -5.760000000000001 |
| T6-OL | -6.393333333333334 |
| T2-YL | -3.9500000000000015 |
| T6-YL | -3.9666666666666663 |
| T6-OL | -3.763333333333333 |
| T2 | -5.570000000000001 |
| T3 | -8.376666666666667 |
| T6 | -12.159999999999998 |
| T2 | -5.946666666666665 |
| T3 | -5.593333333333334 |
| T6 | -12.36 |
### Chart: MAM-Bra029355
| Category | Mam1-pr50 |
|---|---|
| T2-YL | -3.943333333333334 |
| T6-YL | -6.6433333333333335 |
| T6-OL | -6.686666666666667 |
| T2-YL | -3.866666666666667 |
| T6-YL | -3.7666666666666657 |
| T6-OL | -4.916666666666665 |
| T2 | -8.166666666666666 |
| T3 | -8.700000000000001 |
| T6 | -15.14 |
| T2 | -6.1433333333333335 |
| T3 | -4.946666666666666 |
| T6 | -11.646666666666667 || leaf | | Turnip core | |
| --- | --- | --- | --- |
| FT-004 | FT-086 | FT-004 | FT-086 |
| leaf | | Turnip core | |
| --- | --- | --- | --- |
| FT-004 | FT-086 | FT-004 | FT-086 |
### Chart: MAM-Bra029356
| Category | mam3 pr57 |
|---|---|
| T2-YL | -3.013333333333333 |
| T6-YL | -5.45 |
| T6-OL | -5.873333333333334 |
| T2-YL | -3.2400000000000007 |
| T6-YL | -3.633333333333333 |
| T6-OL | -3.6699999999999995 |
| T2 | -5.3500000000000005 |
| T3 | -8.023333333333333 |
| T6 | -16.153333333333332 |
| T2 | -5.329999999999999 |
| T3 | -4.876666666666668 |
| T6 | -12.316666666666663 |
### Chart: MAM-Bra018524
| Category | mam1 pr47 |
|---|---|
| T2-YL | -4.243333333333333 |
| T6-YL | -7.2633333333333345 |
| T6-OL | -7.823333333333334 |
| T2-YL | -4.396666666666667 |
| T6-YL | -5.243333333333332 |
| T6-OL | -5.6066666666666665 |
| T2 | -8.966666666666667 |
| T3 | -10.136666666666668 |
| T6 | -11.423333333333332 |
| T2 | -8.44 |
| T3 | -6.686666666666667 |
| T6 | -11.339999999999998 || leaf | | Turnip core | |
| --- | --- | --- | --- |
| FT-004 | FT-086 | FT-004 | FT-086 |
### Chart: MAM-Bra021947
| Category | mam3 pr56 |
|---|---|
| T2-YL | -3.7599999999999993 |
| T6-YL | -5.486666666666667 |
| T6-OL | -6.100000000000001 |
| T2-YL | -3.426666666666667 |
| T6-YL | -3.91 |
| T6-OL | -3.819999999999999 |
| T2 | -5.54 |
| T3 | -8.17 |
| T6 | -16.846666666666668 |
| T2 | -5.496666666666666 |
| T3 | -4.946666666666668 |
| T6 | -12.856666666666664 || leaf | | Turnip core | |
| --- | --- | --- | --- |
| FT-004 | FT-086 | FT-004 | FT-086 |
Supplemental Fig. S3 Gene expression analysis of MAM paralogues in selected turnip tissues and timepoints in accessions FT-004 and FT-086. Indicated are the expression levels relative to the reference gene (actin) on a logarithmic scale. Error bars indicate standard deviations (n = 3). YL: young leaf; OL: old leaf. For timepoints see legend Fig. 3.
| leaf | | Turnip core | |
| --- | --- | --- | --- |
| FT-004 | FT-086 | FT-004 | FT-086 |
