# Supplementary material for: Glucosinolate variability between turnip organs during development
Source: PLoS One. 2019 Jun 6;14(6):e0217862. doi: 10.1371/journal.pone.0217862 (PMC6553741; doi:10.1371/journal.pone.0217862)
Supplement: S4 Fig — (PPTX) [file pone.0217862.s010.pptx]

## Slide 1
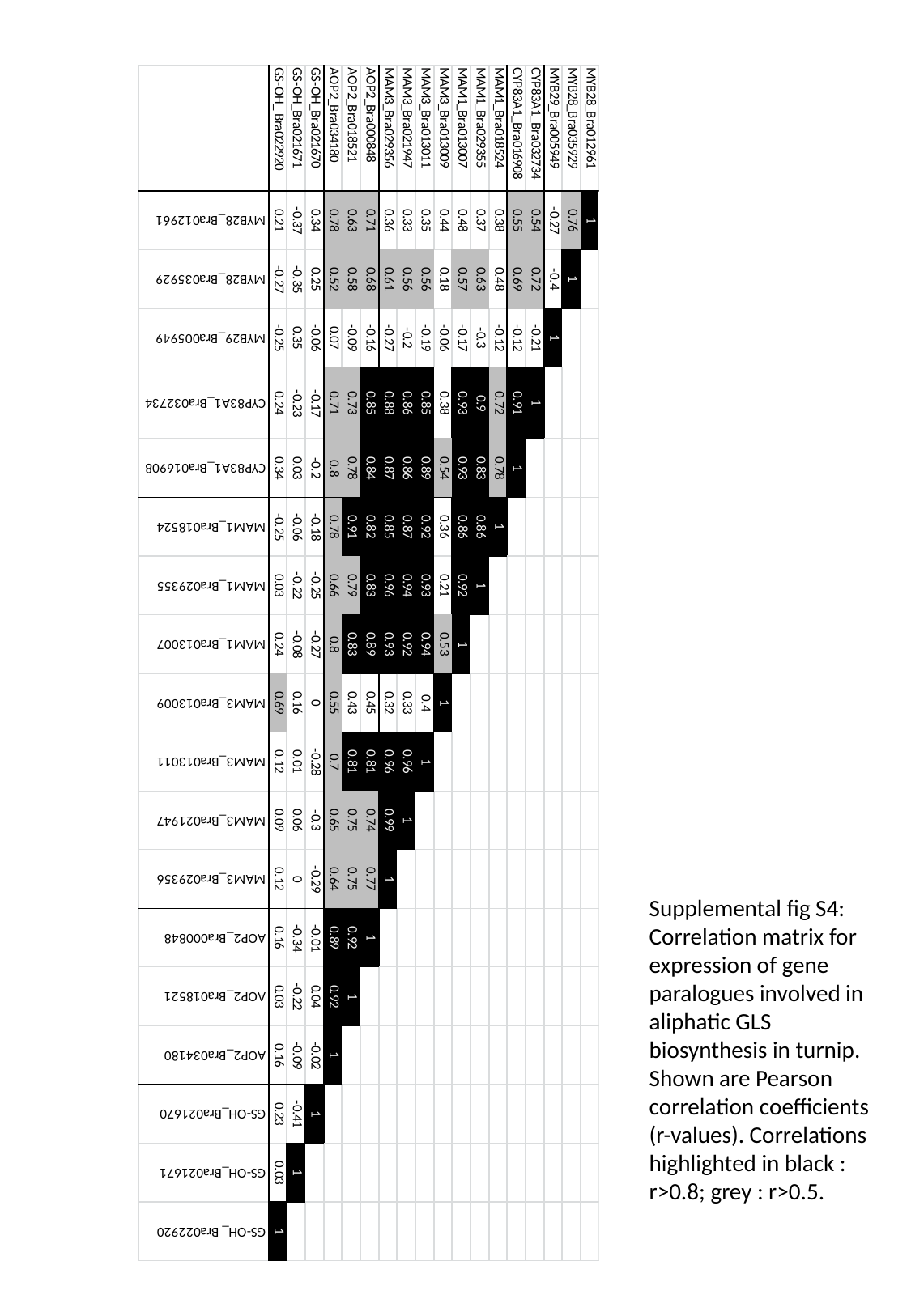

Supplemental fig S4: Correlation matrix for expression of gene paralogues involved in aliphatic GLS biosynthesis in turnip. Shown are Pearson correlation coefficients (r-values). Correlations highlighted in black : r>0.8; grey : r>0.5.
