# Supplementary material for: Glucosinolate variability between turnip organs during development
Source: PLoS One. 2019 Jun 6;14(6):e0217862. doi: 10.1371/journal.pone.0217862 (PMC6553741; doi:10.1371/journal.pone.0217862)
Supplement: S5 Fig — (PPTX) [file pone.0217862.s011.pptx]

## Slide 1
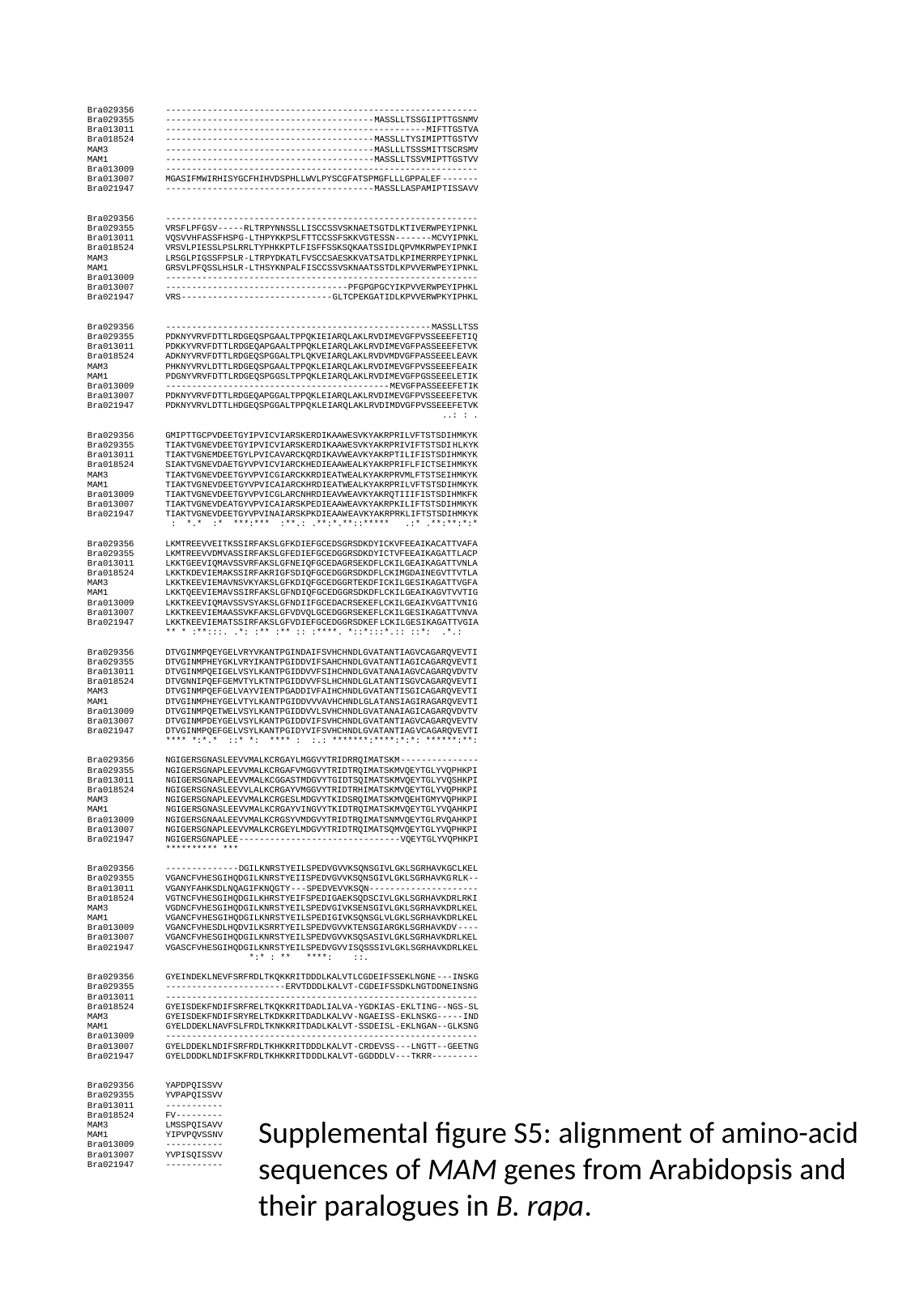

Supplemental figure S5: alignment of amino-acid sequences of MAM genes from Arabidopsis and their paralogues in B. rapa.
